# Entrapment of Hydrophilic and Hydrophobic Molecules in Beads Prepared from Isolated Denatured Whey Protein

**DOI:** 10.3390/pharmaceutics13071001

**Published:** 2021-07-01

**Authors:** Joanne Heade, Robert Kent, Sinead B. Bleiel, David J. Brayden

**Affiliations:** 1 UCD School of Veterinary Medicine and UCD Conway Institute, University College Dublin, Belfield, Dublin 4, Ireland; joanne.heade@ucdconnect.ie; 2 Insucaps LTD., 11 Herbert Street, Dublin, Dublin 2, Ireland; sinead.bleiel@anabio.ie; 3 AnaBio Technologies Ltd., IDA Business Park, Co. Cork, T45 RW24 Carrigtwohill, Ireland; robert.kent@anabio.ie

**Keywords:** Whey protein, denatured whey, oral drug delivery, encapsulation, intestinal absorption

## Abstract

The oral route of administration is by far the most convenient route, especially in the treatment of chronic conditions. However, many therapeutics present formulation difficulties which make them unsuitable for oral delivery. Recently, we synthesized a denatured whey protein isolate (dWPI) bead entrapped with insulin. Our present goal was to assess the suitability of this delivery system to the delivery of other potential molecules, both hydrophilic and hydrophobic. Beads of 1.2–1.5 mm in diameter were entrapped with four payloads representing a range of solubilities. The water-soluble payloads were sodium fluorescein (SF) and FITC dextran 4000 Da (FD4), while the hydrophobic ones were Fast Green and curcumin. Encapsulation efficiency (EE) was 73%, 84%, 70%, and 83% for SF, FD4, Fast Green, and curcumin-loaded beads, respectively. The corresponding loading capacity for each bead was 0.07%, 1.1%, 0.75%, and 1.1%, respectively. Each payload produced different release profiles in simulated gastric fluid (SGF) and simulated intestinal fluids (SIF). SF released steadily in both SGF and SIF. FD4 and curcumin release was not substantial in any buffers, while Fast Green release was low in SGF and high in SIF. The differences in release behaviour were likely due to the varying properties of the payloads. The effect of proteolysis on beads suggested that enzymatic degradation of the whey bead may promote payload release. The beads swelled rapidly in SGF compared to SIF, which likely contributed to the release from the beads, which was largely governed by solvent diffusion and polymer relaxation. Our results offer a systematic examination of the behaviour of hydrophilic and hydrophobic payloads in a dWPI delivery system. These beads may be further designed to orally deliver poorly permeable macromolecules and poorly soluble small molecules of pharmaceutical interest.

## 1. Introduction

Delivery of therapeutics via the oral route is preferred by patients, but it presents formulation difficulties for poorly permeable Biopharmaceutical Class System (BCS) Class III small molecules, peptides and proteins, as well as poorly soluble BCS Class II small molecules. The chief concerns are susceptibility to degradation, solubility, and/or poor permeability at the intestinal epithelium [1]. The gastrointestinal tract (GIT) is a harsh environment which is designed to degrade ingested protein-based substances to generate small peptide nutrients and to eliminate foreign substances. The abundance of proteolytic enzymes is particularly challenging for peptide-based therapeutics [2]. For hydrophobic small molecules or peptides, solubility in gastric and intestinal fluids can be a challenging obstacle for intestinal absorption. It is estimated that 40% of approved drugs and ~90% of drug in development are poorly water soluble [3], creating a great need for delivery systems that can facilitate absorption for drugs with these properties. The final barrier to absorption is the intestinal epithelium which is selectively permeable and can prevent sufficient absorption of some therapeutics including BCS Class II molecules, peptides and other macromolecules. High molecular weight peptides are typically too large and hydrophilic to permeate this barrier [4]. Therapeutics which are too hydrophobic though can also show limited permeability by partitioning into the plasma membrane lipid bilayer too strongly [5].

Numerous strategies have been investigated in attempts to overcome obstacles to oral drug delivery, including the use of micro and nanocarriers, protease inhibitors, permeation enhancers (PE), chemical modifications, excipients to aid solubility, and use of ingestible microneedle and patch devices [6]. While the strategy employed depends largely on the physicochemical properties of the therapeutic, the use of food-grade, natural polymers as delivery vehicles is a promising option for oral delivery. These polymers are low-cost (often by-products from the food industry), biocompatible, and can easily be eliminated from the body [7]. Some examples of such polymers include alginate, pectin, starch, chitosan, and gelatin. Many studies report on their use to develop novel delivery systems for sensitive payloads. Pectin, for example, has been used in an enteric-coated, colon targeted, microbead for the delivery of a tumour suppressor gene in colorectal cancer [8]. In vivo studies showed that the microbeads could deliver intact *p*DNA to rat colon specifically, and that it could be transfected effectively [8]. Another example is a chitosan and alginate nanoparticle for the oral delivery of insulin, which achieved an oral bioavailability of 7% in diabetic rats [9].

Another interesting polymer is whey protein, a by-product of cheese manufacturing. Whey protein isolate (WPI) is used as an encapsulation matrix in micro and nanoparticles because of its capacity for gel formation. These gels can be formed by cold-set gelation, which does not subject the cargo to harsh temperatures or solvents, making it ideal for labile therapeutics [10]. Cold-set gelation requires the WPI to undergo denaturation before the gelation step, which gives it the structural arrangement necessary to form gels either in the presence of salts or by changing the pH [11]. Denatured WPI (dWPI) is reported to have the added benefit of being mucoadhesive [12], having the capacity for protease inhibition, and can act as an intestinal PE [13]. These properties may be particularly beneficial for delivering oral peptides where enzymatic degradation and poor permeability are the two issues to overcome. dWPI-based delivery systems have the potential to be versatile as dWPI contains both hydrophilic and hydrophobic residues [14]. This could be useful in aiding the solubility of BCS Class II small molecule therapeutics. The capacity for hydrophobic interactions was demonstrated by incubating dWPI microbeads in cranberry juice over 3 h (Doherty et al., 2012), where the hydrophobic anthocyanin pigment bound to the dWPI microbead.

WPI has also been shown to improve the storage stability of payloads and resistance to oxidation [15]. The hydrophobic small molecule, curcumin, for example, is quickly degraded at physiological pH and is also sensitive to light [16]. However, it exhibits increased stability against oxidation in the presence of protein [16]. Solghi et al. demonstrated a 60% improvement in oxidative stability when curcumin was encapsulated in dWPI nanoparticles [15].

dWPI has also been used to encapsulate riboflavin (Log P: −1.46) [17], and vitamin D3 (Log P: 7.5) [18] in microbeads and nanoparticles, respectively. We recently showed that dWPI-based beads could be used to encapsulate insulin and that these beads had the capacity to reduce blood glucose levels in rats when administered into intact jejunal loops [19]. Following this, and given the amphiphilic nature of dWPI, we hypothesised that the same delivery system could effectively encapsulate a range of payloads irrespective of aqueous solubility and molecular weight. Using a cold-set, acid-induced gelation method, our aim was to assess the applicability of the delivery system to a broader range of molecule types, which has never been done before for a whey-based delivery system. To achieve this aim, we investigated the capacity of dWPI beads to encapsulate four payloads of varying molecular weights and solubility and we determined the specific effects on the characteristics of the delivery system, i.e., the bead morphology, encapsulation efficiency, loading, release profiles, and release mechanisms.

## 2. Materials and Methods

### 2.1. Materials

BiPro WPI (97%) was obtained from Davisco Foods International Inc. (Eden Prairie, MN, USA), Aerosil^®^-200F was a gift from Evonik (Essen, Germany). Simulated intestinal fluid (SIF) powder was purchased from Biorelevant (London, UK). All other reagents were purchased from Sigma Aldrich (Arklow, Ireland) unless stated otherwise.

### 2.2. Denaturation of Whey Protein Isolate (WPI)

Denaturation of WPI was carried out in advance of acid-induced gelation using our previous method [17]. In this process, 11% (*w*/*v*) WPI was dissolved in HPLC grade water overnight. Following this, the WPI was denatured by stirring in a 78 °C water bath over 45 min. The solution was then rapidly cooled on ice for 1 h followed by overnight storage at 4 °C. The resulting dWPI was used over 2 days.

Denaturation was confirmed by measuring the thiol content of native (nWPI) and denatured WPI as it has been reported that it increases after denaturation [20]. The concentration of free thiol in nWPI and dWPI samples was determined using Ellman’s reagent [21], following the manufacturer’s instructions (Thermo Fisher Scientific, Waltham, MA, USA). Ellman’s reagent was prepared by dissolving 8 mg of the reagent with 2 mL reaction buffer (0.1 M sodium phosphate with 1 mM EDTA). A series of cysteine standards were used to generate a standard curve to determine the thiol content of samples. In this process, 7.9 mg cysteine hydrochloride monohydrate was dissolved in 30 mL reaction buffer (1.5 mM), and 1.25, 1.0, 0.75, 0.5-, and 0.25-mM standards were then made from this stock. nWPI and dWPI were prepared as described earlier and diluted to 1000 μg/mL in reaction buffer. Then, 125 μL of the cysteine standards or WPI samples were mixed with 1250 μL reaction buffer and 25 μL Ellman’s reagent. Samples were incubated at room temperature for 15 min before they were pipetted in duplicate onto a clear flat-bottom 96-well plate. The absorbance was measured at 412 nm and thiol content of samples was calculated using the standard curve. Data for this assay can be found in the Supplementary Materials (Figure S1).

### 2.3. Preparation of Loaded and Unloaded Silica-Coated dWPI Beads

The dWPI was combined under gentle stirring with four separate payloads with each chosen according to MW or solubility. The water-soluble payloads were sodium fluorescein (SF, MW: 376.3 g/mol, Log P: −0.32) [22], and fluorescein isothiocyanate dextran 4000 Da (FD4, MW: 4000 g/mol, Log P: −0.75)). The hydrophobic payloads with low aqueous solubility included Fast Green (MW: 808.9 g/mol, Log P: 2.97), and curcumin (MW: 368.4 g/mol, Log P 3.2). In each case, this yielded a protein-payload mixture that contained 9.9% (*w*/*v*) dWPI and 0.01% (*w*/*v*) payload in the case of SF, or 0.1% (*w*/*v*) payload for FD4, Fast Green, and curcumin. Each payload was dissolved in a solvent prior to incorporation with dWPI: water for SF and FD4, ethanol (33% *v*/*v*) for Fast Green, and methanol (100% *v*/*v*) for curcumin). After ~15 min stirring, beads were produced by adding the mixture dropwise to a sodium acetate hardening solution (pH 4.6) using a 1 mL plastic syringe (Air-Tite™, All-Plastic Henke-Ject™, Fisher Scientific, Hampton, NH, USA) with a 30G needle (Microlance, Becton Dickinson, NJ, USA). This solution was prepared by combining equal volumes of 0.5 M acetic acid and 0.5 M sodium acetate, then adding Tween^®^-20 (0.5% *v*/*v*) to lower the surface tension. The beads were cured for 15 min before rinsing with deionized water several times with the aid of a nylon filter with 50 μm pores (Biodesign™, CellMicroSieves™, Fisher Scientific, Hampton, NH, USA). Beads were then transferred to a Petri dish and air-dried for 1 h to remove surface water. Amorphous fumed silica (Aerosil^®^-200F Evonik, Darmstadt, Germany) (4% wet weight) was then added to the Petri dish and the beads were stirred to allow the coating to take place and left overnight in a fume hood to air-dry. Aerosil^®^-200F was used to adsorb water and prevent the beads from sticking to one another. Once completely dry, the Aerosil^®^-200F was sieved off using a wire mesh sieve (pore size 300 μm). The sieved Aerosil^®^-200F was collected and weighed to monitor the amount of silica that attached to the beads. Unloaded beads were made in the same manner using water in place of a payload. The theoretical loading for each payload was calculated according to Equation (1).

(1) $$\mathrm{Theoretical}\;\mathrm{loading}\;\left(\%\right)=\frac{\mathrm{Total}\;\mathrm{payload}\;\left(\mathrm{mg}\right)}{\mathrm{Total}\;\mathrm{dWPI}+\mathrm{Total}\;\mathrm{payload}\;\left(\mathrm{mg}\right)}\times 100$$

where Total payload and Total dWPI are the amount of payload and dWPI used in the protein–payload mixture.

### 2.4. Sizing and Imaging of dWPI Beads

Standard images of loaded and unloaded dWPI beads for sizing were taken with a Google Pixel smartphone, and the beads were sized using Image J^®^ software. High-quality images of beads were also taken using a stereomicroscope (SZN-T, Optika Srl., Ponteranica, Italy).

### 2.5. Swelling of Unloaded dWPI Beads in Simulated Gastrointestinal Fluids

Dry unloaded beads were weighed and placed into a series of Eppendorf tubes. Then, 1 mL simulated gastric fluid (SGF, pH 1.2) was added to one set of tubes and 1 mL SIF (pH 6.8) was added to a second set. Both simulated fluids were prepared according to United States Pharmacopoeia (USP) specifications (Table 1). Both sets of Eppendorf tubes containing beads and simulated fluids were incubated on a shaker at 600 rpm at 37 °C for 2 h (Titramax 1000, Heidolph, Schwabach, Germany). T_0_ samples were immediately removed, the SGF/SIF was discarded, and the beads were placed on a paper towel before being weighed. This was repeated for all time points up to 2 h. The degree of swelling was calculated according to Equation (2).

(2) $$\mathrm{Degree}\;\mathrm{of}\;\mathrm{Swelling}\;\left(\%\right)=\frac{\mathrm{Weight}\;\mathrm{at}\;\mathrm{Tx}-\mathrm{Initial}\;\mathrm{weight}\;\left(\mathrm{mg}\right)}{\mathrm{Initial}\;\mathrm{weight}\;\left(\mathrm{mg}\right)}$$

where Tx is the designated time point, and the initial weight is the known weight of beads added to that tube. Swelling calculated for T0 samples was subtracted from the other time points to account for any surface water that was not removed by the paper towel. Swelling behaviour in fasted-state simulated gastric fluid (FaSSGF, pH 1.8), and fasted-state simulated intestinal fluid (FaSSIF, pH 6.5) were also examined. These buffers were prepared using SIF powder according to the manufacturer’s instructions (Biorelevant, London, UK) and the composition is provided (Table 1).

### 2.6. Loading and Encapsulation Efficiency of Payloads in dWPI Beads

Loaded beads were ground using a mortar and pestle and the resulting powder was added to 25 mL SIF. For curcumin, the powder was added to 25 mL methanol due to its low solubility in SIF. These solutions were left being stirred vigorously for 4–6 h until the powders had completely dissolved. Then, 200 μL samples were added to a 96-well plate (Corning™, Thermo Fisher Scientific, Waltham, MA, USA) along with a buffer control and analysed. SF and FD4 were analysed using a spectrofluorimeter (MD SpectraMAX^®^ Gemini, San Jose, CA, USA) at an excitation of 490 nm and an emission of 525 nm, while curcumin was analysed at an excitation of 420 nm and an emission of 535 nm. Fast Green was analysed using a spectrophotometer (Biochrom^®^ Asys UVM 340, Cambridge, UK) at 628 nm. Concentrations were calculated from calibration curves for each payload. Encapsulation efficiency (EE), loading, and final loading were calculated according to Equations (3)–(5) below.

(3) $$\mathrm{EE}\;\left(\%\right)=\frac{\mathrm{Total}\;\mathrm{encapsulated}\;\mathrm{payload}\;\left(\mathrm{mg}\right)}{\mathrm{Total}\;\mathrm{payload}-\mathrm{waste}\;\left(\mathrm{mg}\right)}x\;100$$

(4) $$\mathrm{Loading}\;\left(\%\right)=\frac{\mathrm{Total}\;\mathrm{encapsulated}\;\mathrm{payload}\;\left(\mathrm{mg}\right)}{\mathrm{Total}\;\mathrm{beads}\;\left(\mathrm{mg}\right)}x\;100$$

(5) $$\mathrm{Final}\;\mathrm{loading}\;\left(µg/\mathrm{mg}\right)=\frac{\mathrm{Total}\;\mathrm{encapsulated}\;\mathrm{payload}\;\left(µg\right)}{\mathrm{Total}\;\mathrm{beads}\;\left(\mathrm{mg}\right)}$$

where total encapsulated payload is defined as the quantity of payload mixed with the dWPI before encapsulation, and the waste is defined as the payload content of any dWPI-payload solution remaining after bead production (i.e., the residue inside the syringe or beaker).

### 2.7. Release in Simulated Fluids

The in vitro release of the payloads from the dWPI beads was monitored in SGF and SIF. Using a paddle stirrer set at 200 rpm, beads were stirred for 1 h in SGF and then transferred to SIF for a further 3 h. Samples were withdrawn and replaced with fresh buffer at 0, 30, 60 min (for SGF), and then at 0, 30, 60, 90, 120, and 180 min (for SIF). For FD4 and curcumin samples, 50 μL NaOH (0.1 M) was added to all SGF samples to improve the fluorescent signal. Samples were analysed at the wavelengths specified above and concentrations were calculated using the calibration curves. Cumulative release as a percentage of total bead loading was then calculated. Release data were fitted to mathematical drug release models including zero-order, first order, Higuchi, and Korsmeyer–Peppas using the Microsoft^®^ Excel add-in software, DDSolver [23]. The equations for each model are shown in Supplementary Table S1, and equations used to evaluate the best fit for the models for the release profiles are shown in Supplementary Table S2.

Curcumin release was further evaluated in FaSSGF and FaSSIF. Since low aqueous solubility is an issue for curcumin, these beads were chosen to test in FaSSGF/FaSSIF because bile salts in such buffers can improve the solubility of highly hydrophobic compounds such as curcumin [24]. A second FaSSIF solution was also prepared which contained pancreatin (FaSSIF + p). Since the bead itself is made of protein, peptidase attack on whey could likely assist the releasing of curcumin. A FaSSIF solution with 1% (*w*/*v*) pancreatin (4 × USP) was aliquoted and centrifuged for 30 min at 14,000 rpm at 4 °C. The supernatants were combined into a working stock. The release study was then carried out using a water bath on a hot plate to maintain 37 °C. A beaker containing 25 mL FaSSIF with 3% of the pancreatin working solution was placed in the bath and stirred at 200 rpm using a paddle stirrer. This method was modified from Presas et al. [25]. Once the temperature reached 37 °C, curcumin-loaded beads were added to the beaker. A T_0_ sample was immediately withdrawn and replaced with FaSSIF containing 3% of the pancreatin working solution. Samples were taken at 0, 15, 30, 60, 120, 180, and 240 min and pipetted into a black 96-well plate. As with the SGF samples, NaOH was added to FaSSGF samples and they were analysed at a wavelength of 420 ex, 565 em (nm). FaSSIF samples were read at a wavelength of 420 ex, 535 em (nm). A protein assay was carried out on the FaSSIF + P solution to monitor the pancreatin concentration. This was performed using a Quant-iT™ protein assay kit according to the manufacturer’s instructions (Thermo Fisher Scientific, Waltham, MA, USA).

### 2.8. Statistical Analysis

One-way ANOVA with a Bonferroni post-test was used to compare EE and loading for each of the payloads. The swelling study and release data for curcumin were analysed by 2-way ANOVA with Bonferroni’s post-test. Data were presented as mean ± the standard deviation (SD). Results were considered significant if *p* < 0.05 compared to control. Statistical analysis was carried out using GraphPad^®^ Prism Version 5.0 (San Diego, CA, USA).

## 3. Results

### 3.1. Swelling of Dried Unloaded Silica-Coated dWPI Beads

**Figure 1:**
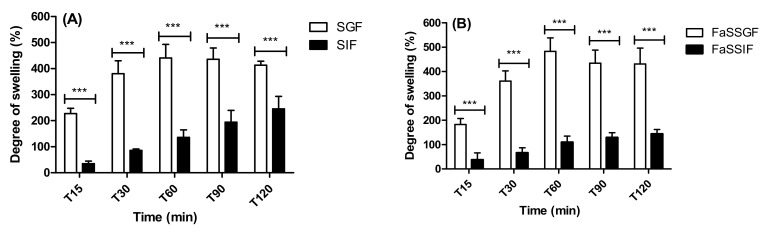
The swelling behaviour as indicated by weight changes of unloaded beads in (**A**) SGF/SIF and, (**B**) FaSSGF/FaSSIF. Data are presented as mean ± SD, asterisks indicate significance between the simulated fluids (**A**) between SGF and SIF, and (**B**) between FaSSGF and FaSSIF) at the respective time point. *** *p* > 0.001, (*n* = 3 batches).

The swelling study showed a significant difference in weight between unloaded beads incubated in SGF (pH 1.2) and SIF (pH 6.8) (Figure 1A), and also between FaSSGF (pH 1.6) and FaSSIF (pH 6.5) (Figure 1B). It should be noted that the SIF and FaSSIF data are only relevant for small intestinal instillation or for an orally delivered enteric-coated system where the effects of stomach acid are negated. This is because non-enteric coated beads arriving in the small intestinal fluid would have already been swollen from their passage through the gastric fluid. The beads absorbed water rapidly at the acidic pH values of SGF and FaSSGF, absorbing ~200% of their initial weight within 15 min. In contrast to this, the bead swelling in SIF and FaSSIF was less dramatic and more gradual over the 2 h. There were no differences in bead weights between incubation in SGF and FaSSGF (Supplementary Figure S2). There was, however, a significant difference between SIF and FaSSIF at 120 min, suggesting that minor differences in pH did not alter swelling at gastric pH but may change bead behaviour at intestinal pH.

### 3.2. Encapsulation Efficiency, Loading, Diameter, and Morphology of Loaded dWPI Beads

**Figure 2:**
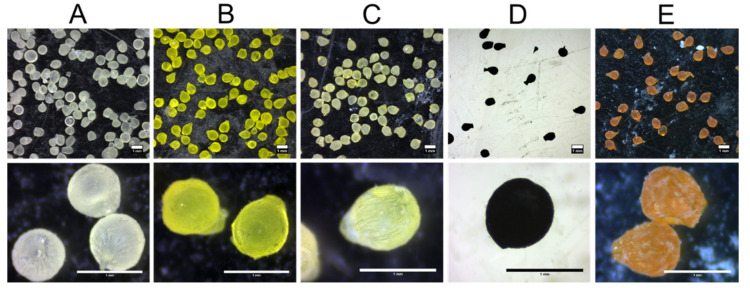
Stereo micrographs of (**A**) unloaded beads, and beads loaded with (**B**) SF, (**C**) FD4, (**D**) Fast Green, and (**E**) curcumin. Horizontal scale bars = 1 mm.

SF, FD4, Fast Green and curcumin-loaded beads had similarly high EE values (70–84%) and moderate loading values (0.07–1.1%) (Table 2 There were no significant differences between loaded bead types, except for SF-loaded beads. These beads showed a lower loading (0.07%) compared to the other payloads because a lower concentration of SF was used in the formulation for detection purposes, as it is a much more intensely fluorescent molecule. Overall, the loading values were high relative to the theoretical loadings, though lower than other delivery systems made from natural polymers [9,25,26]. Still, the beads may achieve a higher payload capacity if the payload concentration were increased. The average size of the SF-loaded beads (1.49 ± 0.44 mm) was statistically larger than beads loaded with the other payloads. SF and unloaded beads, which were of a similar size (1.40 ± 0.04 mm), were the first batches produced. Since there was no difference in size between beads loaded with FD4, Fast Green, and curcumin, it is possible that the bead size reduced as operator proficiency in synthesis increased. Figure 2 shows unloaded and loaded beads under different magnifications. The shape of the beads is primarily spherical, but some tear-shaped beads can be seen and some with flat edges. The higher magnification shows the frosted surface of the beads caused by the presence of Aerosil^®^ on the surface. Ridges can also be seen on the surface of the beads at the higher magnification, along with indents (Figure 2E) that could have been formed from beads being in contact with one another during drying. The addition of payloads to the beads did not alter the morphology to any great extent. However, a higher frequency of tailing was observed in the Fast Green-loaded beads (Figure 2D) and curcumin loaded beads (Figure 2E), both of which contained an organic solvent in the formulation rather than water; this may have affected gelling.

### 3.3. The Release Profile of Payloads in Simulated Fluids

**Figure 3:**
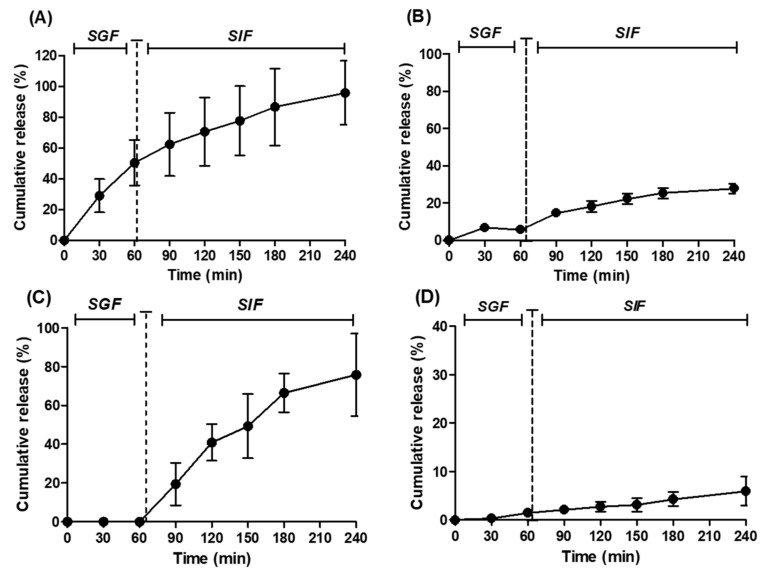
Release profiles in SGF and SIF from dWPI beads loaded with: (**A**) SF, (**B**) FD4, (**C**) Fast Green, and (**D**) curcumin. Vertical discontinuous bars indicate the switch of buffer from SGF to SIF. Each point represents the mean ± SD (*n* = 3 batches for all).

SF beads quickly released 50% of payloads in SGF over 1 h, followed by a slow continuous release of the remainder over 3 h in SIF (Figure 3A). The release profile for FD4 did not follow the same pattern as SF. Very little FD4 was detected after 1 h in SGF and only ~25% was released over the 3 h in SIF (Figure 3B). This suggests that the release of FD4 from beads might be related to the change in pH or perhaps the high molecular weight slowed release. No Fast Green release was observed in SGF, but there was steady and rapid release in SIF (Figure 3C). ~80% Fast Green was released over 3 h in SIF. Little release was observed for the curcumin beads in SGF/SIF. Approximately 6% of the encapsulated curcumin was released over the 4 h, with only 1% of this occurring in SGF (Figure 3D). The Korsmeyer–Peppas model was the best fit for the SF, Fast Green, and curcumin beads (Supplementary Table S3). In this model, the diffusional constant (*n*) describes the release mechanism. For spherical particles when 0.43 < *n* < 0.85 the release is non-Fickian anomalous transport. This is when diffusion and polymer relaxation occur to the same extent [27]. This was the case for SF-loaded beads. When *n* > 0.85 for spherical particles, release followed non-Fickian Super Case II transport. Fast Green and curcumin release fell into this category. In this type of release, the diffusion rate is higher than polymer relaxation and the solvent penetrates the particles at an accelerated rate [27]. FD4 release fits best with the first order release model which suggests that its release is concentration-dependent. The *n* values of the Korsmeyer–Peppas model also indicate the release kinetics are haracterized by diffusion and polymer relaxation [27]. Though these models only give an indication of a mechanism, these data suggest that solvent diffusion and polymer relaxation contribute substantially to the payload release.

**Figure 4:**
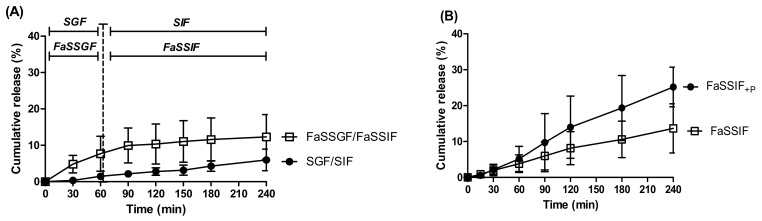
Release of curcumin from dWPI beads in: (**A**) SGF/SIF and FaSSGF/FaSSIF. Vertical discontinuous bar indicates switch of buffer from SGF/FaSSGF to SIF/FaSSIF, respectively. (**B**) FaSSIF + P and FaSSIF at 37 °C. Each point represents the mean ± SD (*n* = 3 batches).

Curcumin beads were further examined in FaSSGF and FaSSIF to see if the presence of bile salts would aid solubility and increase release (Figure 4A). No statistical difference was found between curcumin release in SGF/SIF and FaSSGF/FaSSIF, although on average, more curcumin was released in FaSSGF/FaSSIF than in the former combination (12% compared to 6% in SGF/SIF). Most of the release occurred in FaSSGF (7%), which has a lower concentration of bile salts compared to FaSSIF, suggesting that the bile salts did not aid release or that acidic pH had a greater impact. After 1 h in FaSSGF, the release rate plateaued in FaSSIF and after the 3 h, only ~10% of the curcumin had been released in total. The next step in mimicking the small intestinal environment was to add proteolytic enzymes—60 μg/mL pancreatin was added to FaSSIF to create FaSSIF + P (as measured by the protein assay) and release was carried out over 4 h at 37 °C. The results were variable and there was no significant difference between the two groups; however, there was a trend suggesting that beads released ~12% more curcumin in FaSSIF + P than in FaSSIF (Figure 4B). Overall, little curcumin was released, regardless of the buffer used.

## 4. Discussion

Encapsulation of peptides and other unstable molecules in delivery vehicles is a common strategy used in oral drug delivery to overcome low solubility, proteolysis, poor intestinal permeability, and to improve storage stability. The milk-derived protein, dWPI, has multiple functional properties that make it an ideal encapsulation matrix for oral delivery of unstable cargos. Oral peptide formulations, in particular, can benefit from the dWPI-based oral delivery system as it may offer mucoadhesion, enzyme inhibition, and permeation enhancement without the need for additional excipients. dWPI beads encapsulate a range of molecules with different properties, and this is demonstrated in our study. All of the payloads used here showed high encapsulation efficiency and good loading irrespective of molecular weight and aqueous solubility. Of the characteristics examined, release from the beads was the characteristic most affected by the payload properties. This is particularly interesting as therapeutics with similar physiochemical properties could behave in a comparable way to the payloads used in this study. This work could provide a starting point in the development of dWPI-based formulations by giving an insight into the characteristics that could be observed for similar payloads. The development of effective oral formulations for unstable bioactive molecules is a long and risky process and our study could provide valuable information which may prevent costly failures.

The Korsmeyer–Peppas model of drug release suggested that solvent diffusion and polymer relaxation were important steps in the bead-release mechanism. These events are also necessary for swelling to occur [28]. This means that swelling likely facilitates release and the pattern observed could be a good indication of release behaviour. Unloaded beads displayed a greater degree of swelling at pH 1.2 compared to pH 6.8 which could have been an indication that, for some payloads, there will be substantial release in the stomach. This swelling pattern was also observed for our insulin-loaded dWPI beads [19], which also displayed release in acidic buffer. In the present study, SF (water-soluble, low molecular weight) was the only payload to release rapidly at low pH. It is possible that SF may not have strongly interacted with the dWPI and that it diffused from beads upon swelling initiation. For bioactive molecules that target the small intestine and might behave similarly to SF, an enteric-coating or gel capsule could prevent gastric release and improve delivery. This rate of swelling in SIF might also facilitate mucoadhesion in the small intestine, which is often the target for oral therapeutics [29].

None of the other three payloads demonstrated gastric release to any great extent. This could suggest that there were other factors involved in the release, but an examination of the swelling behaviour of these beads could help elucidate the release pattern. FD4 (hydrophilic, high molecular weight) release from the beads was much slower than sodium fluorescein, which could be due to the molecular weight or perhaps an interaction between the payload and dWPI. It has been reported that dextran and dWPI can form covalent bonds [30,31], though FTIR analysis would be needed to confirm this was the case for these beads. Fast Green showed no release at pH 1.2 followed by a quick release at pH 6.8. Fast Green is a stain that forms electrostatic interactions with proteins [32]. It is possible the shift in pH when the beads were moved to SIF weakened the interaction and liberated the Fast Green. Curcumin showed very little release in SGF and SIF, which could be due to its hydrophobicity and solubility issues. The study did not show any improvement in curcumin release with the addition of bile salts to the FaSSIF, but there was an indication that proteolysis of dWPI could facilitate release. Despite this result, BCS Class II molecules in general have been shown to benefit from increased stability and dissolution in WPI formulations [33,34]. Though the stability of the beads and payloads were not investigated in this study, we have previously shown that this dWPI delivery system has the capacity to protect encapsulated insulin from degradation for 60 min in SIF supplemented with pancreatin [19].

The release characteristics presented here are an example of how hydrophilic and hydrophobic therapeutics may behave when released from dWPI beads. However, another benefit of dWPI-based beads is that they are highly modifiable. Release patterns in the small intestine can be altered by changing the composition and thickness of coatings [35]. The drying method is another factor that could be changed to alter the bead properties. As we demonstrated with our insulin-loaded dWPI beads, lyophilisation can increase the release rate by increasing porosity of the beads [19,36]. Another benefit of this dWPI-based delivery system is that it has good scalability which we demonstrated by using a Buchi B-390 encapsulator (BÜCHI Labortechnik, Flawil, Switzerland) to produce insulin-loaded beads [19]. Difficulty in scaling can be an obstacle in the transition from a bench-top delivery system to a manufacturable system relevant to the pharmaceutical industry. Factors that need to be considered are the cost and hazards associated with the materials, the time required for production, and the reproducibility of key characteristics of the delivery system [37]. However, dWPI beads are made from cheap, non-toxic, and readily available diary ingredients, and can be made in large quantities and in a range of bead sizes using industrial methods.

## 5. Conclusions

This dWPI-based delivery system is a low-cost and versatile option for the oral delivery of a range of molecules. The delivery system is applicable to both hydrophilic and hydrophobic compounds, and the payloads examined here were encapsulated with high encapsulation efficiency and reasonable loading regardless of their physiochemical properties. Examination of bead release and release kinetics revealed that the payload properties affected the release behaviour of the beads more than they affected any other bead characteristic. This release was also likely facilitated by bead swelling. There was also some evidence to suggest that enzymatic degradation of the beads in the intestine may promote further payload release. This delivery system has the potential to be useful in the oral delivery of hydrophilic and hydrophobic therapeutics.

## Figures and Tables

**Table 1:** The composition of the five simulated fluids used in this study.

	SGF	SIF	FaSSGF	FaSSIF	FaSSIF_+P_
NaCl (mM)	34		34	106	106
KH_2_PO_4_ (mM)	-	49	-	-	-
NaOH pellets (mM)	-	-	-	10.5	10.5
NaH_2_PO_4_ (mM)	-	-	-	29	29
Taurocholate (mM)	-	-	0.08	3	3
Lecithin (mM)	-	-	0.02	0.75	0.75
Pancreatin (µg/mL)	-	-	-	-	60 ± 6
pH	1.2	6.8	1.8	6.5	6.5

Taurocholate and lecithin were added to FaSSGF, FaSSIF, and FaSSIF_+p_ in the form of SIF Powder (Biorelevant, London, UK). FaSSIF_+p_: FaSSIF with pancreatin.

**Table 2:** Theoretical, actual, and final loading of beads along with EE and diameters. Data are presented as mean ± SD, (*n* = 3 for each). * *p* < 0.05 compared to the three other payloads.

Payload	Theoretical Loading (%, *w*/*w*)	EE (%)	Loading (%)	Final loading (µg/mg)	Diameter (mm)
SF	0.1	72.62 ± 3.79	0.07 ± 0.003 *	0.72 ± 0.04 *	1.49 ± 0.44 *
FD4	1.0	83.59 ± 17.67	1.13 ± 0.23	11.33 ± 2.27	1.31 ± 0.37
Fast Green	1.0	69.79 ± 10.62	0.75 ± 0.04	7.49 ± 0.42	1.22 ± 0.27
Curcumin	1.0	82.99 ± 15.65	1.09 ± 0.15	10.89 ± 1.51	1.29 ± 0.31
Unloaded	-	-	-	-	1.40 ± 0.04

## Data Availability

The data presented in this study are available on reasonable request from the corresponding author.

## Supplementary Materials

The following are available online at https://www.mdpi.com/article/10.3390/pharmaceutics13071001/s1, Figure S1. The free thiol concentration in nWPI and dWPI. Data is presented as mean ± SEM, *** *p* > 0.001 (n = 3). Figure S2. A comparison of the swelling behaviour of unloaded beads in (A) SGF/FaSSGF and, (B) SIF/FaSSIF. Data is presented as mean ± SD, asterisks indicate significance between the simulated fluids (SIF and FaSSIF) at the time point indicated. ** *p* > 0.01, (n = 3 batches). Table S1. The formulas used for each mathematical model of drug release. F = fraction (%) of drug released over time (t). Adapted from Zhang et al. [23]. Table S2. The formulas used to calculate R^2^adj and AIC. These parameters were used to find the model with the best fit for each release profile. Considering both values is appropriate when comparing models with different numbers of parameters, as described by Zhang et al. [23]. Table S3. The R^2^adj and other parameters calculated for each release model applied to the SGF/SIF release data. Highlighted values in grey indicate the best fit (highest R^2^adj and the lowest AIC).

## References

[1] Brayden D.J., Hill T.A., Fairlie D.P., Maher S., Mrsny R.J. (2020). Systemic Delivery of Peptides by the Oral Route: Formulation and Medicinal Chemistry Approaches. Adv. Drug Deliv. Rev..

[2] Hamman J.H., Enslin G.M., Kotzé A.F. (2005). Oral delivery of peptide drugs: Barriers and developments. BioDrugs.

[3] Loftsson T., Brewster M.E. (2010). Pharmaceutical applications of cyclodextrins: Basic science and product development. J. Pharm Pharm..

[4] Cano-Cebrián M.J., Zornoza T., Granero L., Polache A. (2005). Intestinal absorption enhancement via the paracellular route by fatty acids, chitosans and others: A target for drug delivery. Curr. Drug Deliv..

[5] Boyd B.J., Bergström C.A.S., Vinarov Z., Kuentz M., Brouwers J., Augustijns P., Brandl M., Bernkop-Schnürch A., Shrestha N., Préat V. (2019). Successful oral delivery of poorly water-soluble drugs both depends on the intraluminal behavior of drugs and of appropriate advanced drug delivery systems. Eur. J. Pharm. Sci..

[6] Mantaj J., Vllasaliu D. (2020). Recent advances in the oral delivery of biologics. Pharm. J..

[7] Idrees H., Zaidi S.Z.J., Sabir A., Khan R.U., Zhang X., Hassan S.U. (2020). A Review of Biodegradable Natural Polymer-Based Nanoparticles for Drug Delivery Applications. Nanomaterials.

[8] Bhatt P., Khatri N., Kumar M., Baradia D., Misra A. (2015). Microbeads mediated oral plasmid DNA delivery using polymethacrylate vectors: An effectual groundwork for colorectal cancer. Drug Deliv..

[9] Sarmento B., Ribeiro A., Veiga F., Sampaio P., Neufeld R., Ferreira D. (2007). Alginate/chitosan nanoparticles are effective for oral insulin delivery. Pharm. Res..

[10] Doherty S.B., Gee V.L., Ross R.P., Stanton C., Fitzgerald G.F., Brodborb A. (2011). Development and characterisation of whey protein micro-beads as potential matrices for probiotic protection. Food Hydrocoll..

[11] Alting A.C., Hamer R.J., de Kruif C.G., Visschers R.W. (2000). Formation of disulfide bonds in acid-induced gels of preheated whey protein isolate. J. Agric. Food Chem..

[12] Hsein H., Garrait G., Beyssac E., Hoffart V. (2015). Whey protein mucoadhesive properties for oral drug delivery: Mucin-whey protein interaction and mucoadhesive bond strength. Colloids Surf. B Biointerfaces.

[13] Déat-Lainé E., Hoffart V., Garrait G., Beyssac E. (2013). Whey protein and alginate hydrogel microparticles for insulin intestinal absorption: Evaluation of permeability enhancement properties on Caco-2 cells. Int. J. Pharm..

[14] Dissanayake M., Vasiljevic T. (2009). Functional properties of whey proteins affected by heat treatment and hydrodynamic high-pressure shearing. J. Dairy Sci..

[15] Solghi S., Emam-Djomeh Z., Fathi M., Farahani F. (2020). The encapsulation of curcumin by whey protein: Assessment of the stability and bioactivity. J. Food Process. Eng..

[16] Schneider C., Gordon O.N., Edwards R.L., Luis P.B. (2015). Degradation of Curcumin: From Mechanism to Biological Implications. J. Agric. Food Chem..

[17] O’Neill G.J., Egan T., Jacquier J.C., O’Sullivan M., Dolores O’Riordan E. (2014). Whey microbeads as a matrix for the encapsulation and immobilisation of riboflavin and peptides. Food Chem..

[18] Abbasi A., Emam-Djomeh Z., Mousavi M., Davoodi D. (2014). Stability of vitamin D3 encapsulated in nanoparticles of whey protein isolate. Food Chem..

[19] Heade J., McCartney F., Chenlo M., Marro O.M., Severic M., Kent R., Bleiel S.B., Alvarez C.V., Griffin B.T., Brayden D.J. (2021). Synthesis and In Vivo Evaluation of Insulin-Loaded Whey Beads as an Oral Peptide Delivery System. Pharmaceutics.

[20] Hsein H., Garrait G., Mumin M.A., Beyssac E., Hoffart V. (2017). Atomization of denatured whey proteins as a novel and simple way to improve oral drug delivery system properties. Int. J. Biol. Macromol..

[21] Aitken A., Learmonth M., Walker J.M. (2009). Estimation of disulfide bonds using Ellman’s reagent. The Protein Protocols Handbook.

[22] Pellosi D.S., Estevão B.M., Semensato J., Severino D., Baptista M.S., Politi M.J., Hioka N., Caetano W. (2012). Photophysical properties and interactions of xanthene dyes in aqueous micelles. J. Photochem. Photobiol. A.

[23] Zhang Y., Huo M., Zhou J., Zou A., Li W., Yao C., Xie S. (2010). DDSolver: An add-in program for modeling and comparison of drug dissolution profiles. AAPS J..

[24] Pavlović N., Goločorbin-Kon S., Ðanić M., Stanimirov B., Al-Salami H., Stankov K., Mikov M. (2018). Bile acids and their derivatives as potential modifiers of drug release and pharmacokinetic profiles. Front. Pharmacol..

[25] Presas E., McCartney F., Sultan E., Hunger C., Nellen S., Alvarez C.V., Werner U., Bazile D., Brayden D.J., O’Driscoll C.M. (2018). Physicochemical, pharmacokinetic and pharmacodynamic analyses of amphiphilic cyclodextrin-based nanoparticles designed to enhance intestinal delivery of insulin. J. Control. Release.

[26] Sahoo P., Hoong Leong K., Nyamathulla S., Onuki Y., Takayama K., Yong Chung L. (2019). Chitosan complexed carboxymethylated iota-carrageenan oral insulin particles: Stability, permeability and in vivo evaluation. Mater. Today Commun..

[27] Bruschi M.L. (2015). Mathematical models of drug release. Strategies to Modify the Drug Release from Pharmaceutical Systems.

[28] Ostrowska-Czubenko J., Gierszewska M., Pieróg M. (2015). pH-responsive hydrogel membranes based on modified chitosan: Water transport and kinetics of swelling. J. Polym. Res..

[29] Güler M.A., Gök M.K., Figen A.K., Özgümüş S. (2015). Swelling, mechanical and mucoadhesion properties of Mt/starch-g-PMAA nanocomposite hydrogels. Appl. Clay Sci..

[30] Turan D., Gibis M., Gunes G., Baier S.K., Weiss J. (2018). The impact of the molecular weight of dextran on formation of whey protein isolate (WPI)-dextran conjugates in fibers produced by needleless electrospinning after annealing. Food Funct..

[31] Zhu D., Damodaran S., Lucey J.A. (2008). Formation of whey protein isolate (WPI)-dextran conjugates in aqueous solutions. J. Agric. Food Chem..

[32] Corradini M.G., Melton L., Shahidi F., Varelis P. (2019). Synthetic food colors. Encyclopedia of Food Chemistry.

[33] Geng T., Banerjee P., Lu Z., Zoghbi A., Li T., Wang B. (2017). Comparative study on stabilizing ability of food protein, non-ionic surfactant and anionic surfactant on BCS type II drug carvedilol loaded nanosuspension: Physicochemical and pharmacokinetic investigation. Eur. J. Pharm. Sci..

[34] Taghavi Kevij H., Mohammadian M., Salami M. (2019). Complexation of curcumin with whey protein isolate for enhancing its aqueous solubility through a solvent-free pH-driven approach. J. Food Process. Preserv..

[35] Doherty S.B., Auty M.A., Stanton C., Ross R.P., Fitzgerald G.F., Brodkorb A. (2012). Application of whey protein micro-bead coatings for enhanced strength and probiotic protection during fruit juice storage and gastric incubation. J. Microencapsul..

[36] Fonte P., Soares S., Costa A., Andrade J.C., Seabra V., Reis S., Sarmento B. (2012). Effect of cryoprotectants on the porosity and stability of insulin-loaded PLGA nanoparticles after freeze-drying. Biomatter.

[37] Paliwal R., Babu R.J., Palakurthi S. (2014). Nanomedicine scale-up technologies: Feasibilities and challenges. AAPS PharmSciTech.

