# Supplementary material for: Entrapment of Hydrophilic and Hydrophobic Molecules in Beads Prepared from Isolated Denatured Whey Protein
Source: Pharmaceutics. 2021 Jul 1;13(7):1001. doi: 10.3390/pharmaceutics13071001 (PMC8309121; doi:10.3390/pharmaceutics13071001)
Supplement: Supplementary file 1 [file pharmaceutics-13-01001-s001.zip › pharmaceutics-1256892- supplementary.pdf]

pharmaceutics

MDPI

Supplementary Materials: Entrapment of Hydrophilic and Hydrophobic Molecules in Beads Prepared from Isolated Denatured Whey Protein

Joanne Heade, Robert Kent, Sinead B. Bleiel, and David J. Brayden

S1. Determination of denaturation

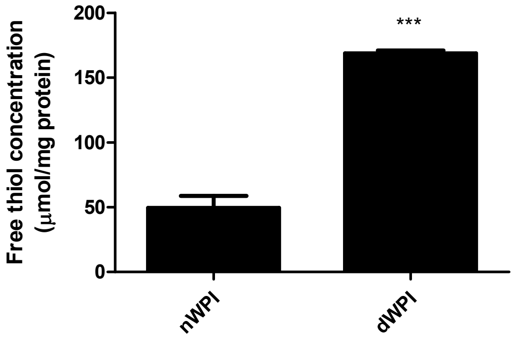

Figure S1. The free thiol concentration in nWPI and dWPI. Data is presented as mean ± SEM, ***P > 0.001 (n = 3).

S2. Swelling behavior

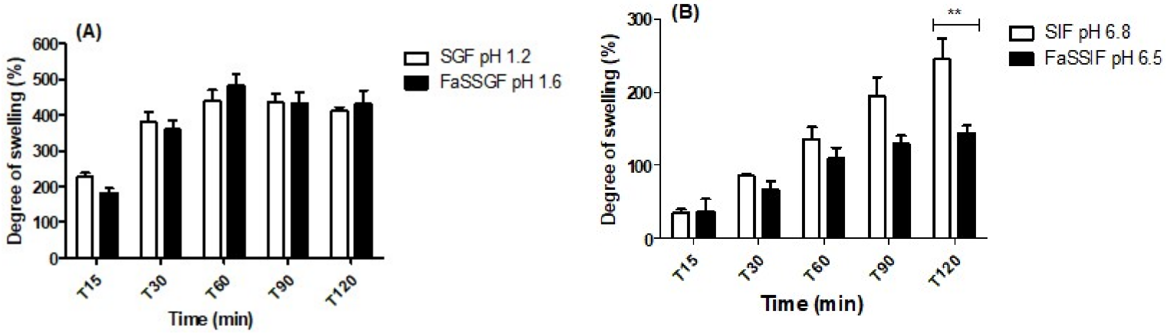

Figure S2. A comparison of the swelling behaviour of unloaded beads in (A) SGF / FaSSGF and, (B) SIF / FaSSIF. Data is presented as mean ± SD, asterisks indicate significance between the simulated fluids (SIF and FaSSIF) at the time point indicated. **P > 0.01, (n = 3 batches).

S3. Release modelling

Table S1. The formulas used for each mathematical model of drug release. F = fraction (%) of drug released over time (t). Adapted from Zhang et al. [23].

Model	Formula	Parameters
Zero Order	$F = k_0(t)$	k_0 = zero order release constant
First Order	$F = 100(1 - e^{-k1(t)})$	k_1 = first order release constant
Higuchi	$F = k_H(t^{0.5})$	k_H = Higushi release constant
Korsmeyer-Peppas	$F = k_{KP}(t^n)$	k_{KP} = release constant incorporating structural and geometric characteristics of the drug-dosage form<br>n = diffusional exponent

Table S2. The formulas used to calculate R^2adj and AIC. These parameters were used to find the model with the best fit for each release profile. Considering both values is appropriate when comparing models with different numbers of parameters, as described by Zhang et al. [23].

Equation	Formula	Parameters
R^2 adjusted	$R^2adj = 1 - \frac{n-1}{n-p}(1 - R^2)$	n = number of data points<br>P = number of parameters
Akaike Information Criterion	$AIC = (n)In(WSS) + 2(p)$	n = number of data points<br>P = number of parameters<br>WSS = weighted sum of squares

Table S3. The R^2adj and other parameters calculated for each release model applied to the SGF/SIF release data. Highlighted values in grey indicate the best fit (highest R^2adj and the lowest AIC).

	Parameters	SF	FD4	Fast Green	Curcumin
Zero order	k_0	0.489	0.134	0.317	0.024
	R^2adj	0.7752	0.9224	0.8893	0.9290
	AIC	59.7923	33.4488	54.3286	4.3666
First order	k_1 (min^{-1})	0.012	0.002	0.004	0.000
	R^2adj	0.972	0.946	0.815	0.928
	AIC	40.580	30.723	58.737	4.741
Higuchi	k_H	6.333	1.686	3.795	0.291
	R^2adj	0.9836	0.8899	0.6728	0.8065
	AIC	39.2469	36.7881	63.3704	13.4063
Korsmeyer-Peppas	kKP	6.275	0.439	0.065	0.026
	n	0.528	0.439	1.311	1.083
	R^2adj	0.989	0.789	0.904	0.957
	AIC	36.295	31.788	53.616	1.3520

R^2adj = R^2 adjusted, AIC = Akaike Information Criterion
